# A Next Generation Sequencing approach to the mutational screening of patients affected with systemic autoinflammatory disorders: diagnosis improvement and interpretation of complex clinical phenotypes

**DOI:** 10.1186/1546-0096-13-S1-O24

**Published:** 2015-09-28

**Authors:** M Rusmini, S Federici, F Caroli, A Grossi, M Baldi, L Obici, A Insalaco, A Tommasini, R Caorsi, E Gallo, AN Olivieri, AV Marzano, D Coviello, R Ravazzolo, A Martini, M Gattorno, I Ceccherini

**Affiliations:** 1 IRCCS G Gaslini, UOC Medical Genetics, Genoa, Italy; 2 IRCCS G Gaslini, UOC Pediatric Rheumatology, Genoa, Italy; 3 Ospedale Galliera, Laboratory of Human Genetics, Genoa, Italy; 4 IRCCS Policlinico S Matteo, Laboratorio Biotecnologie, Pavia, Italy; 5 Bambin Gesù Childern's Hospital, Division of Rheumatology, Rome, Italy; 6 IRCCS Burlo Garofolo, Department of Pediatrics, Trieste, Italy; 7 IRCCS G Gaslini, Department of Pediatrics, Genoa, Italy; 8 Università di Torino, Dipartimento di Salute Pubblica e Pediatria, Torino, Italy; 9 Second University of Naples, Women, Child and General and Specialistic Surgery, Naples, Italy; 10 Università degli studi di Milano, UO Dermatologia, Milan, Italy; 11 IRCCS, UOC Pediatric Rheumatology, Genoa, Italy

## Introduction

Systemic autoinflammatory diseases (SAIDs) are a group of monogenic disorders characterized by inflammation which occurs in the absence of pathogenic auto-antibodies, auto-reactive T lymphocytes or other infective causes. More than 50% of SAID patients recruited to our Unit does not show any mutation at gene(s) tested by direct Sanger sequencing in the routine diagnosis. Clinical misdiagnosis, mutations in untested gene regions and genetic heterogeneity are possible explanations.

## Objectives

To improve both the molecular diagnosis and genotype interpretation of SAIDs, we aimed at the development of a Next Generation Sequencing based protocol, designed for the simultaneous screening of ten genes known to be involved in a remarkable proportion of SAIDs. Different bio-informatics approaches were taken into consideration in order to define the best pipeline for variants detection.

## Patients and methods

A panel of gene amplicons specific for the diseases under study was designed through the Ion AmpliSeq™ designer software. Fifty SAID patients, already genotyped for the respective causative gene(s), were massively sequenced for the coding portions of MEFV, MVK, TNFRSF1A, NLRP3, NLRP12, NOD2, PSTPIP1, IL1RN, LPIN2 and PSMB8. Three different bio-informatics pipelines, Ion Reporter™, CLC Bio Genomics Workbench, and GATK-based in-house workflow, were compared

## Results

The approach we propose here for NGS-based diagnosis of SAIDs, has resulted technically suitable, with a very high mean coverage (336X) and nearly full detection of variants. Besides expected mutation, we could also identify many unexpected variants that were all validated by Sanger sequencing and compared to assess true and false positive detection rates of the three workflows. Finally, the overall clinical picture of 34 patients were re-evaluated in the light of the new mutations found.

## Conclusions

The present gene panel has resulted suitable for molecular diagnosis of SAIDs. Besides causative mutations, most patients have turned out to carry variants of unclear significance that will need further investigation. Moreover, genotype-phenotype correlation drawn in 34 patients has confirmed a remarkably difficult interpretation of NGS data in patients with an undefined or complex inflammatory phenotype. This supports the need of evidence-based and validated clinical criteria as crucial tools to be used concurrently with the genetic analysis for the final diagnosis and classification of SAIDs patients. In addition to setting a first approach to mutational detection in SAIDs, patients left without a clear diagnosis will be identified as candidate for the successive NGS analysis of the whole exome.

